# Incidence and Predictors of Maternal and Perinatal Mortality among Women with Severe Maternal Outcomes: A Tanzanian Facility-Based Survey for Improving Maternal and Newborn Care

**DOI:** 10.1155/2020/5390903

**Published:** 2020-04-10

**Authors:** Athanase Lilungulu, Deogratius Bintabara, Simon Mujungu, Enid Chiwanga, Paulo Chetto, Mzee Nassoro

**Affiliations:** ^1^Department of Obstetrics and Gynaecology, College of Health Sciences, The University of Dodoma, Dodoma, Tanzania; ^2^Department of Public Health, College of Health Sciences, The University of Dodoma, Dodoma, Tanzania; ^3^Department of Obstetrics and Gynaecology, Dodoma Regional Referral Hospital, Dodoma, Tanzania

## Abstract

**Introduction:**

Maternal and perinatal mortality is still a major public health challenge in Tanzania, despite the ongoing government efforts to improve maternal and newborn care. Among the contributors to these problems is the high magnitude of severe maternal outcomes (maternal near-miss). The current study, therefore, aimed to identify the magnitude and predictors of maternal and perinatal mortality among women with severe maternal outcomes admitted to Dodoma Regional Referral Hospital.

**Methods:**

A retrospective cross-sectional study was conducted from October 2015 to January 2016 at Dodoma Regional Referral Hospital in Dodoma City. All maternal deaths and maternal near-misses based on WHO criteria were included in this study. Three outcome variables have been identified: maternal mortality, perinatal mortality, and neonatal complications. To examine the predictors for the three predetermined outcome variables, the three logit models each containing unadjusted and adjusted findings were fitted. A *P*-value less than 0.05 was considered indicative of statistically significant.

**Results:**

A total of 3600 pregnant women were admitted for obstetric reasons during the mentioned period. 140 of them were diagnosed with severe maternal outcomes; hence, they were included in this study. The severe maternal outcome incidence ratio was 40.23 per 1000 live births, the institutional maternal mortality ratio was 459.77 per 100000 live births, and the perinatal mortality rate was 10.83 per 1000 total births. Most of the maternal morbidity and mortality were due to direct causes in which postpartum hemorrhage and hypertensive disorders were the leading causes. In adjusted analysis, per-protocol management, maternal age, and mode of birth were predictors of maternal mortality, perinatal mortality, and neonatal complications, respectively.

**Conclusion:**

Establishing and strengthening obstetric ICUs will help reduce maternal mortality as the response time from the onset of obstetric complications, while the provision of high-quality care will be substantially reduced. Furthermore, the study recommends regular provision of in-service refresher training to emphasize the practice and compliance of per-protocol case management through a team approach in order to reduce the burden of maternal and perinatal mortality in Tanzania.

## 1. Introduction

Maternal and perinatal mortality are the crucial indicators used globally to assess, monitor, and evaluate maternal and newborn health [1], and countries have made improvements towards the achievement of Sustainable Development Goals (SDGs) [2]. These two events are rare in high-income countries but more experienced in low- and middle-income countries (LMICs), which contribute nearly 99% and 98% of all maternal and perinatal deaths worldwide, respectively [3, 4]. Of these deaths, 66% are contributed by sub-Saharan Africa (SSA) alone [5]. Recent research studies conducted in Tanzania highlighted a notable surge of maternal mortality ratio (MMR) (from 454 to 556 maternal deaths per 100 000 live births), while perinatal mortality ratio (PMR) slightly increased (36–39 perinatal deaths per 1000 all total births) between 2010 and 2016 [6, 7]. This incremental is related to some deficiencies in recognizing and managing obstetric complications that range from minor morbidity to life-threatening conditions which need attention [8, 9].

Women with life-threatening conditions may end up having severe maternal outcomes (SMOs), which are either maternal death (MD) or maternal near-miss (MNM) [10]. According to the World Health Organization (WHO), MD refers to the death of a woman while pregnant or within 42 days of termination of pregnancy, irrespective of the duration, and site of the pregnancy, from any cause related to or aggravated by the pregnancy or its management but not from accidental or incidental causes [10]. On the other hand, MNM defines MD as a woman who nearly died but survived a complication that occurred during pregnancy, childbirth, or within 42 days of the termination of pregnancy. In practical terms, women are considered near-miss cases when they survive life-threatening conditions (i.e., organ dysfunction) [10, 11]. Despite MD and MNM having two separate outcomes, MNM almost always accounts for the most characteristics of MD. Equally, in many cases, it tends to occur immediately before MD [8, 12]. Studying all of them under the same umbrella will, therefore, increase the in-depth understanding of the issues behind maternal and newborn health in a low-resource setting such as Tanzania.

In response to the burden of maternal and perinatal deaths, the government of Tanzania through its Ministry of Health attempted to improve maternal and child health services. It managed to increase the coverage of antenatal care (ANC) provided by skilled providers (96%–98%), institutional birth (50%–63%), and births assisted by skilled attendants (51%–64%) between 2010 and 2016 [6, 7]. However, concerns have been raised regarding the poor quality of care which has been cited as the major barrier to the reduction of mortalities [13]. Therefore, despite reported increase in utilization of maternal health services, the findings from two different research studies conducted in two different regions in Tanzania revealed that the country is still experiencing a high number of women with SMO [14, 15]. In addition, these studies highlighted the magnitude, incidences, and potential factors that could lead to MNM. Unfortunately, they did not assess the magnitude and predictors of perinatal deaths at the same time as the results of SMO. So far, there is limited information and lack of studies that assessed the predictors of both maternal and perinatal deaths among women at the same time with SMO.

A better understanding of the predictors of both maternal and perinatal deaths among women with SMO is critical for the country to achieve global targets for preventable maternal and perinatal mortality (<140 maternal deaths per 100 000 live births, ≤12 neonatal deaths per 1000 live births, and ≤12 per 1000 total births) by 2030 [16]. As such, by using the WHO criteria for recognizing women with MNM, the current study aimed at identifying the magnitude and predictors of maternal and perinatal mortality among women with SMO admitted to Dodoma Regional Referral Hospital in central Tanzania. The results from the current study will lay the groundwork for the administrators, policy-makers, and researchers to strengthen maternal and newborn care in Tanzania and other countries with similar settings.

## 2. Materials and Methods

### 2.1. Study Design Setting

A retrospective cross-sectional study was conducted among pregnant women with SMO in the obstetrics and gynecology ward at Dodoma Regional Referral Hospital (DRRH), central Tanzania. This hospital provides 24-hour birth services as well as emergency obstetric care for all pregnant women. The hospital is also used as the teaching hospital for both undergraduate and postgraduate students from the College of Health Sciences, The University of Dodoma, Tanzania.

### 2.2. Study Population

All pregnant women who were admitted and diagnosed to have SMO or maternal deaths due to SMO based on the WHO definition [17, 18] from October 2015 to January 2016 were targeted as study participants.

### 2.3. Sample Size and Sampling Procedure

The sample size was obtained by using the formula for a single population proportion. A sample size of 141 pregnant women with SMO was calculated by employing the following assumptions during sample size determination: the proportion of pregnant women who deliver within the hospitals and experience MNM in low-resource settings was 8.23% [19], the level of significance was 95%, the margin of error was 5%, and by considering a nonresponse rate was 20%.

**Figure 1:**
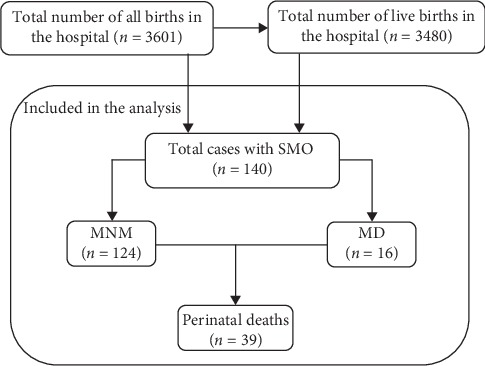
Flowchart of sampling units included in this analysis.

All women with SMO (MNM and MD) were eligible and included in this study. However, all pregnancy loss with a gestational age of less than 28 weeks were excluded. Accordingly, during the study period, a total of 3601 pregnant women with a gestational age of 28 weeks and above were admitted and delivered; of which, 140 (99.7% response rate) met the inclusion criteria. Their information regarding morbidity, mortality, and pregnancy outcomes was collected. For details of the sample and sampling, see Figure 1.

## 3. Measurement of Variables

### 3.1. Outcome Variables

In this study, three outcome variables have been identified: maternal mortality, perinatal mortality, and neonatal complications. The first outcome variable was maternal mortality, which was coded as “Yes” if the women died due to SMO, while those who survived were coded as “No.” The second outcome variable was perinatal mortality, which was coded as “Yes” if the pregnant outcome resulted in either stillbirth or early neonatal deaths, otherwise was coded as “No.” The third outcome variable was neonatal complications which was coded as “Yes” if the baby was born with any complications (SAMM) (congenital anomalies not considered), otherwise was coded as “No.”

### 3.2. Independent Variables

Maternal age was coded “0” for the ages between 15 and 19, “1” for the age between 20 and 34, and “2” for the age of 35 and more. The residence was coded as “Urban” for the women coming from areas located in cities, municipalities, and town councils gazetted under the Local Government Act, 1982 [20], and “Rural” for those who came from the areas that were located outside the urban setting. Parity was coded as “Nulliparous” for women with no history of previous birth, “Para 1–3” for those whose history of the previous birth was one to three, and “Para 4 or more” for those whose history of the previous birth was four or more. ANC visit was coded as “attended” for women who visited ANC at least once before admitted, otherwise was coded as “not attended.” The mode of referral was coded as “self-referral” for the women who came to the hospital directly from home and “facility referral” for women who came to the hospital after being referred from lower facilities. The care receiving time was coded as “immediate” for women who received care less than 30 minutes from the time they arrived at the hospital, otherwise was coded as “late.” Hemorrhagic shock was coded as “mild,” “moderate,” and “severe” for women presented with blood volume loss of approximately “15–30%,” “30–40%,” and “> 40%,” respectively. Blood pressure was coded as “elevated” if the woman presented with systolic BP of ≥130 mmHg or diastolic BP of ≥90 mmHg, otherwise were coded as “normal.” The mode of birth was coded as “surgical intervention” for women who had a caesarean section or assisted vaginal birth and “normal birth” for those who had a spontaneous vaginal birth. Per-protocol management through a team approach was coded “Yes” if a patient was reviewed by a team which includes obstetricians, midwives, neonatologists, and anesthetists as per protocol, otherwise was coded as “No.”

## 4. Operational Definition

### 4.1. Maternal Near-Miss (MNM)

This is an occasion in which a pregnant woman comes close to maternal death but does not die—a “near-miss.”

### 4.2. Maternal Near-Miss Incidence Ratio (MNMIR)

This is the number of maternal near-miss cases per 1000 live births.

### 4.3. Severe Maternal Outcomes (SMOs)

This is the number of all women with either maternal near-miss or maternal death due to severe obstetric complications.

### 4.4. Severe Maternal Outcome Incidence Ratio (SMOIR)

This is the number of all women with either maternal near-miss or maternal death due to severe obstetric complications per 1000 live births.

### 4.5. Maternal Near-Miss Mortality Ratio (MNMMR)

This is the ratio of a number of maternal near-miss cases per maternal deaths.

### 4.6. Institutional Maternal Mortality Ratio (IMMR)

This is the ratio of the number of maternal deaths during a period of four months per 100,000 live births during the same time period.

### 4.7. Case Fatality Rate (CFR)

This is the proportion of a number of maternal deaths among all women with severe maternal outcomes.

### 4.8. Perinatal Morbidity Rate (PMR)

This is the number of perinatal complications and deaths per 1000 total births.

### 4.9. Perinatal Mortality Rate (PNMR)

This is the number of perinatal deaths per 1000 total births.

## 5. Data Source and Collection

All the data of women with SMO between the aforementioned periods were directly identified and extracted from the database which was updated daily. For the cases that met the inclusion criteria, three authors used a structured data abstraction questionnaire to collect the information. The medical files were, thereafter, retrieved for detailed information regarding diagnosis, management, and outcome of the selected cases. To confirm the diagnosis, three authors independently reviewed the medical files and reached a consensus on whether the diagnosis of MNM was correct or not.

## 6. Statistical Analysis

The data were analyzed using Stata 14 (StataCorp, College Station, TX). In descriptive analyses, categorical variables were summarized using proportions and then presented in tables and graphs, while continuous variables were summarized using mean (SD). To examine the predictors for the three predetermined outcome variables, the three logit models, each containing unadjusted and adjusted findings, were fitted. For each model, we first fitted the unadjusted models (1a, 2a, and 3a), and all variables which showed association at *P* < 0.2 were considered to fit the adjusted models (1b, 2b, and 3b), respectively. In all adjusted models, the stepwise (backward elimination) method was used to build the final models. A *t*-test for each of the coefficient in adjusted models was calculated and used to test for the association. A *P*-value of less than 0.05 was considered indicative of statistically significant association.

## 7. Ethical Considerations

Ethical clearance was approved by the University of Dodoma (UDOM) Research Ethics Committee. Permission was also obtained from DRRH administration. Autonomy was observed, and confidentiality and clinical documentation assured that the information obtained would not be exposed to any nonconcerned member.

## 8. Results

### 8.1. Baseline Characteristics of the Subjects

A total of 3601 pregnant women were admitted for obstetric reasons during the four months of the study period; of which 3480 gave live births while 140 were diagnosed with SMO and then included in this study.  Table 1 presents a summary of the baseline characteristics of the included study participants. The mean age of the respondents (SD) was 26.9 (6.5) years, and an average number of birth per women (SD) was 1.8 (1.7) deliveries. About one-third (34.3%) of the respondents were nulliparous. The majority (87, 62.14%) of the patients were referred from lower-level health facilities. In addition, the majority (88, 62.86%) presented either moderate or severe hemorrhagic shock. However, less than 40% received obstetric emergency care within 30 minutes from the time they arrived at the hospital.

### 8.2. Major Services or Intervention provided to the Patients with SMO

**Figure 2:**
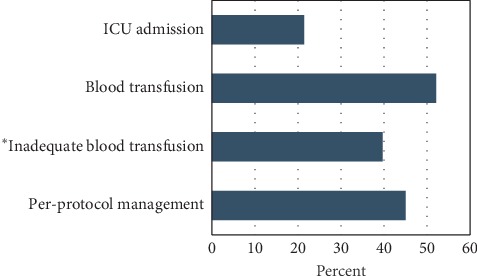
Major intervention/service provided for pregnant women with SMO, DRRH, Tanzania, January 2016, (*n* = 140). *∗n* = 73, the total number of subjects who received blood transfusion.

Of the all the SMO subjects, less than a quarter (21.4%) were admitted to the intensive care unit (ICU) for close care and follow-up while nearly half (45.0%) received per-protocol management from skilled health professionals team. On the other hand, 73 (52.1%) of the subjects required a blood transfusion. However, nearly 40% of them received an inadequate amount of the required blood as prescribed by physicians as shown in Figure 2.

### 8.3. Maternal and Perinatal Outcome due to SMO

Table 2 presents maternal and perinatal outcome indicators among women diagnosed to have SMO. The results show that for every 1000 live births, there were about 41 SMOs of which about 36 were MNM, while institutional MMR was about 460 maternal deaths per 100000 live births. Furthermore, for every maternal death in that hospital, there were about eight MNM cases, with an estimated CFR of 11.43%. Regarding the perinatal outcome, for every 1000 births, there were about 11 perinatal deaths.

### 8.4. Underlying Causes of Maternal Morbidity and Mortality

Table 3 presents the magnitude of the direct and indirect underlying causes of maternal morbidity and mortality. The high proportion (92, 65.71%) of maternal morbidity was contributed by direct causes which also were responsible for about two-thirds of total maternal mortality. Among the direct causes, postpartum hemorrhage and hypertensive disorders were the leading causes of both maternal morbidity (17.86% and 17.14%) and mortality (18.75% and 25.00%), respectively. While for the indirect causes, severe anemia was the major cause of both maternal morbidity (17.14%) and mortality (18.75%).

### 8.5. Predictors for Maternal and Perinatal Mortality and Neonatal Complications

Table 4 represents the results of unadjusted (1a, 2a, and 3a) and adjusted (1b, 2b, and 3b) logit models. In adjusted model 1b (maternal mortality), the results showed that women who were admitted to the ICU were more likely to die, while those who had received per-protocol management through a team approach were less likely to die when holding all other selected independent variables constant. Additionally, in model 2b (perinatal mortality), the results showed that women aged 20 or higher were more likely to experience perinatal mortality compared to those with less than 20 years. Furthermore, in model 3b (neonatal complications), newborns delivered through spontaneous vaginal birth and those whose mothers presented with severe hemorrhagic shock were more likely to be born with complications compared to their counterparts.

## 9. Discussion

To the best of our knowledge, the current study is the first in the region to assess the predictors of both maternal and perinatal mortality and neonatal complications among women reported having SMO at DRRH. In this study, we found a high number of women with SMO, high hospital MMR, and PNMR. In addition, most of the maternal morbidity and mortality were due to direct causes, in which postpartum hemorrhage and hypertensive disorders were the leading causes. Furthermore, per-protocol management, maternal age, and mode of birth were predictors of maternal mortality, perinatal mortality, and neonatal complications among women with SMO, respectively.

The relatively high SMO incidence ratio (MD and MNM) observed in the current study shows that there is a weakness in prevention and treatment of obstetric emergencies in the study region despite the known and availability of effective evidenced-based approaches [21, 22]. Similar findings have been reported in earlier studies conducted in other regions within the country [14, 15] and neighboring countries such as Rwanda [23] and Ethiopia [24]. The study also revealed a high proportion of newborns with poor perinatal outcomes as a result of SMO. Similar findings have been reported in the previous study conducted in Nigeria [25]. The similarity of these findings in relation to high SMO and poor perinatal outcomes might be explained by the fact that all these countries are located in SSA, the region known with a high burden of severe maternal morbidity and mortality [2, 26]. Furthermore, the similar factors associated with these problems have been reported among all countries within this region [27].

The existing evidence indicates that the top three direct causes of MD in SSA are obstetric hemorrhage, hypertensive disorders (i.e., eclampsia), and sepsis [28]. The current study buttresses this evidence, as we found out that postpartum hemorrhage and hypertensive disorders are the major leading causes of MD among women with SMO in Tanzania. The persistent high number of women dying due to these conditions might be due to the inadequate availability and readiness of the lower-level and public facilities in Tanzania to provide basic emergency obstetric and newborn care (BEmONC) [29, 30]. Similar findings have been reported in other previous studies conducted in Tanzania [14, 15] and other areas with similar setting [25, 31, 32]. In addition, despite the fact we found out that postpartum hemorrhage was the number one killer among women with SMO, most of the patients received an inadequate amount of the required units of blood as per physicians' directives. This might be due to not only inadequate availability of blood in blood stores across the country but also poor birth preparedness and complications readiness strategy practices which emphasize women to identify two potential blood donors in case of emergency [33].

Maternal morbidity and mortality can largely be reduced by ensuring that the health systems adapt safe motherhood initiatives such as the provision of ICU services for critical obstetric conditions leading to SMO [34]. In this study, less than a quarter of the women with MNM received treatment in the ICU. Furthermore, contrary to our expectations, our findings revealed that women with MNM who were admitted to adult ICU were more likely to die compared to their counterparts. This might be explained by the fact that there is no separate adult ICU for critically ill obstetric patients at DRRH. Together with the reported inadequate availability of BEmONC among Tanzania health facilities [29, 30], these might compromise the provision of ICU services. Equally, in majority of health facilities in Tanzania, adult ICUs have a limited number of patients' beds and only those who are critically ill are admitted. Our findings may, therefore, partly be explained by the low survival rate due to those reasons. In addition, MNM requires aggressive interventions through a team approach involving obstetricians, midwives, neonatologists, intensivists, and anesthetists as per protocol [35]. In this study, we found out that less than half of the patients with SMO received per-protocol case review and management based on hospital context. Using a team approach technique in the management of MNM as per protocol is essential to save a maternal life [36]. In line with this argument, our finding revealed that women with MNM who received per-protocol management with a team approach were less likely to die.

Previous studies indicated that SMO is higher among older women and also more associated with adverse perinatal outcomes [37–39]. The current study went further and revealed that women (20 years and above) were more likely to have poor perinatal outcomes (perinatal mortality) compared to younger women (less than 20 years). Furthermore, the majority of those newborns who survived developed complications. When we assessed the risk factors, we found out that neonatal complications were more likely to occur among women with SMO who had spontaneous vaginal birth. This might be explained by the fact that most women with SMO are critically ill and that such a state may compromise many physiological processes during normal birth. In addition, SMO is associated with neonatal complications along the mode of birth [40]. However, there questions still abound regarding the causal pathway and its interpretations [41]. Our finding is, however, contrary to the normal situation where spontaneous vaginal birth is considered less likely to cause neonatal complications than the surgical mode of birth [42].

The strength of the current study is that it analyzed and presents the data obtained from the tertiary hospital which accommodates all cases of SMO within the region. Therefore, it suggests that our findings reflect the actual situation regarding the burden of SMO and its effect on maternal and perinatal outcomes. However, the study faced some limitations. Notably, one of the major limitations is the missing of some information related to compliance of protocols resulting in assessment based on case management through a team approach only. This might have overestimated the proportion of those who were regarded to have received the per-protocol case management approach. Another limitation was the failure to include the pregnant women with a gestational age of less than 28 weeks since this might have affected the estimates of SMO, MD, MNM, and perinatal deaths. Similarly, the duration of four-month retrospective follow-up of SMO cases was very short and this might have affected the process of calculating maternal and perinatal indicators such as HMMR and PNMR.

## 10. Conclusion

The study revealed that SMO, MMR, and PNMR are major public health problems in Tanzania that threaten maternal and newborn health. Establishing and strengthening obstetric ICUs will help to reduce maternal mortality as the response time from onset of obstetric complications (e.g., eclampsia and hemorrhage) to provision of high-quality care will be substantially reduced. Furthermore, the study recommends regular provision of in-service refresher training to emphasize the practice and compliance of per-protocol case management through a team approach in order to reduce the burden of maternal and perinatal mortality in Tanzania.

## Figures and Tables

**Table 1:** Baseline and clinical characteristics on admission of the subjects, DRRH, Tanzania, January 2016 (*n* = 140).

Variable	*n* (%)
Age in years (mean = 26.86, SD = 6.47)	
15–19	27 (19.29)
20–34	83 (59.28)
≥35	30 (21.43)

Residence	
Rural	59 (49.29)
Urban	71 (50.71)

Parity (mean = 1.79, SD = 1.73)	
Nulliparous	48 (34.29)
Para 1–3	61 (43.57)
Para 4 or more	31 (22.14)

Antenatal care	
Attended	126 (90.00)
Not attended	14 (10.00)

Mode of referral	
Self-referral	53 (37.86)
Facility referral	87 (62.14)

Time to receive care	
Immediate	54 (38.57)
Late	86 (61.43)

Hemorrhagic shock	
None or mild	52 (37.14)
Moderate	58 (41.43)
Severe	30 (21.43)

Blood pressure	
Elevated (systolic or diastolic)	118 (84.29)
Normal	22 (15.71)

Mode of birth	
Surgical intervention	79 (56.43)
Normal delivery	61 (43.57)

**Table 2:** Maternal and perinatal outcomes indicators among pregnant women with SMO, DRRH, Tanzania, January 2016.

Variable	SAMM indicators
All births in the hospital	3601
All live births in the hospital	3480

Severe maternal outcomes (SMOs)	
Maternal deaths (*n*)	16
Maternal near-miss (MNM) (*n*)	124
Total SMO (*n*)	140

Overall SMO indicators	
SMO incidence ratio (per 1000 live births)	40.23
MNM incidence ratio (per 1000 live births)	35.63
Institutional MMR (per 100000 live births)	459.77
MNM mortality ratio	7.75
Case fatality rate (CFR) or mortality index (%)	11.43

Perinatal outcome	
Born with good condition (*n*)	77
Born with complications (*n*)	24
Died (*n*)	39
Percent of poor perinatal outcome (*n* = 63)	45.00

Perinatal indicators	
Perinatal morbidity rate (per 1000 births)	17.50
Perinatal mortality rate (per 1000 total births)	10.83

**Table 3:** Direct and indirect causes of maternal morbidity and mortality among pregnant women with SMO, DRRH, January 2016.

Variable	Morbidity (*n* = 140) *n* (%)	Mortality (*n* = 16) *n* (%)
Direct causes	104 (74.28)	10 (62.5)
Antepartum hemorrhage	20 (14.29)	1 (6.25)
Postpartum hemorrhage	25 (17.86)	3 (18.75)
Hypertensive disorders	24 (17.14)	4 (25.00)
Impending or ruptured uterus	10 (7.14)	1 (6.25)
Obstructed labor	8 (5.72)	0 (0.00)
Puerperal sepsis	12 (8.57)	0 (0.00)
Others	5 (3.57)	1 (6.25)

Indirect causes	36 (25.72)	6 (37.50)
Severe anemia	24 (17.14)	3 (18.75)
Cardiovascular diseases	8 (5.72)	3 (18.75)
HIV/AIDS related	3 (2.14)	0 (0.00)
Malaria	1 (0.71)	0 (0.00)

**Table 4:** Factor associated with maternal and perinatal mortality and neonatal complications, DRRH, Tanzania, January 2016.

Variable	Maternal mortality (model 1)				Perinatal mortality (model 2)				Neonatal complications (model 3)			
	a) Unadjusted *β* (SE)	*P*	b) Adjusted *β* (SE)	*P*	a) Unadjusted *β* (SE)	*P*	b) Adjusted *β* (SE)	*P*	a) Unadjusted *β* (SE)	*P*	b) Adjusted *β* (SE)	*P*
Age in years (ref: <20)												
20–34	1.480 (1.066)	0.165	0.154 (1.291)	0.905	1.684 (0.773)	0.029	1.864 (0.873)	0.033	0.693 (0.620)	0.263		
≥35	1.061 (1.187)	0.371	−1.302 (1.529)	0.394	2.120 (0.824)	0.010	2.724 (1.049)	0.009	0.406 (0.787)	0.606		

Residence (ref: rural)												
Urban	0.251 (0.535)	0.639			0.174 (0.378)	0.645			0.026 (0.468)	0.956		

Parity (ref: nulliparous)												
Para 1–3	2.221 (1.068)	0.038	2.093 (1.246)	0.093	0.464 (0.453)	0.305	−0.376 (0.569)	0.508	−0.446 (0.538)	0.407		
Para 4 or more	2.201 (1.122)	0.049	2.244 (1.399)	0.109	0.737 (0.517)	0.154	−1.153 (0.803)	0.151	0.182 (0.611)	0.766		

Antenatal care (ref: attended)												
Not attended	−0.563 (1.074)	0.600			1.751 (0.596)	0.003	1.314 (0.670)	0.060				

Mode of referral (ref: facility referral												
Self-referral	0.017 (0.548)	0.975			−0.627 (0.383)	0.102	−0.563 (0.456)	0.217				

Mode of birth (ref: surgical)												
Spontaneous vaginal birth	−0.284 (0.547)	0.604			−0.441 (0.389)	0.257			1.088 (0.491)	0.027	1.225 (0.543)	0.024

Time to receive care (ref: <30 minutes												
30 minutes or more	0.707 (0.607)	0.243			0.635 (0.409)	0.120	0.402 (0.578)	0.487	−0.395 (0.470)	0.401		

Elevated BP (ref: no)												
Yes	0.297 (0.794))	0.708			−1.168 (0.478)	0.015	−1.022 (0.551)	0.064	0.376 (0.819)	0.647		

Eclampsia/ preeclampsia (ref: no)												
Yes	0.550 (0.627)	0.380			−1.147 (0.649)	0.077			0.617 (0.538)	0.251		

Hemorrhagic shock (ref: no)												
Moderate	0.499 (0.658)	0.448			−0.454 (0.458)	0.322	0.021 (0.534)	0.968	0.579 (0.598)	0.333	0.885 (0.658)	0.179
Severe	0.875 (0.715)	0.221			0..865 (0.481)	0.072	0.640 (0.548)	0.243	2.138 (0.696)	0.002	1.911 (0.739)	0.010

ICU admission (ref: no)												
Yes	1.534 (0.553)	0.005	1.313 (0.616)	0.033	0.134 (0.452)	0.768			−0.348 (0.613)	0.570		

Per-protocol management (ref: no)												
Yes	−1.914 (0.777)	0.014	−1.920 (0.813)	0.018	−0.520 (0.389)	0.181	0.191 (0.568)	0.736	−0.823 (0.490)	0.093	−0.931 (0.616)	0.131

*β* = estimated beta coefficient; SE = standard errors of the estimated beta coefficient; ref = reference category; *P* = *P* value.

## Data Availability

The data used to support the findings of this study are available from the corresponding author upon request.
